# TMX2-Related Neurodevelopmental Disorder With Bilateral Polymicrogyria and Syndactyly: A Case Report From Morocco

**DOI:** 10.7759/cureus.111145

**Published:** 2026-06-19

**Authors:** Asmae Baaziz, Asmaa Mdaghri Alaoui

**Affiliations:** 1 Pediatrics P2, Children's Hospital, Ibn Sina University Hospital, Rabat, MAR

**Keywords:** autosomal recessive disorder, consanguinity, cortical malformation, epilepsy, microcephaly, neurodevelopmental disorder, polymicrogyria, syndactyly, tmx2, whole-exome sequencing

## Abstract

Thioredoxin-related transmembrane protein 2 (TMX2)-related neurodevelopmental disorder (OMIM #618730) is an exceedingly rare autosomal recessive condition caused by biallelic pathogenic variants in *TMX2*, a gene encoding an endoplasmic reticulum (ER)-resident oxidoreductase involved in neuronal survival and cortical development. The condition is characterized by severe intellectual disability, microcephaly, spasticity, epilepsy, and cortical malformations, primarily polymicrogyria.

We report a 13-month-old Moroccan male infant born to second-cousin consanguineous parents, presenting with severe global developmental delay, profound axial hypotonia, absent head control, and progressive microcephaly (head circumference 41 cm, < -3 standard deviation (SD)). Epilepsy was diagnosed at seven months of age and managed with valproic acid. Clinical examination revealed characteristic dysmorphic features, including microcephaly, a sloping forehead, bilateral strabismus, retrognathism, low-set ears and hairline, and bilateral second-third toe syndactyly - a feature not previously described in association with *TMX2*-related disorder. Brain MRI demonstrated bilateral frontoparietal polymicrogyria. Electroencephalography showed right hemispheric dysfunction with focal epileptic activity. Whole-exome sequencing identified a homozygous pathogenic missense variant in *TMX2* (NM_015959.4:c.614G>A; p.Arg205Gln), classified as pathogenic in ClinVar (VCV000804370), confirming the diagnosis.

This case expands the phenotypic spectrum of *TMX2*-related disorder by documenting bilateral second-third toe syndactyly and highlights the diagnostic value of whole-exome sequencing in consanguineous families presenting with cortical malformations and severe neurodevelopmental delay. Early molecular diagnosis is essential for appropriate genetic counseling and multidisciplinary management.

## Introduction

Thioredoxin-related transmembrane protein 2 (TMX2), OMIM #616718, encodes an endoplasmic reticulum (ER)-resident oxidoreductase belonging to the thioredoxin superfamily. Redox homeostasis refers to the cellular maintenance of a balanced oxidation-reduction state, which is essential for normal protein folding and organelle function. *TMX2 *localizes to mitochondria-associated ER membrane contact sites, where it plays a critical role in maintaining cellular redox homeostasis through regulation of disulfide bond formation and mitochondrial calcium flux [1,2]. Within the developing nervous system, *TMX2 *is highly expressed in neurons and radial glial progenitor cells, where it modulates mitochondrial dynamics and apoptotic pathways critical for cortical neurogenesis. Loss of *TMX2 *function impairs neuronal survival and disrupts radial migration during embryonic brain development, resulting in cortical malformations [1,3].

Biallelic pathogenic variants in *TMX2 *cause a rare autosomal recessive neurodevelopmental disorder (OMIM #618730) characterized by severe developmental delay, intellectual disability, progressive microcephaly, axial hypotonia evolving into spasticity, epilepsy, and cortical malformations, particularly polymicrogyria - a cortical malformation defined by an excessive number of abnormally small gyri with irregular folding of the cerebral cortex, resulting in disrupted cortical architecture and function [1]. Since its initial description by Vandervore et al. in 2019, fewer than 20 cases have been reported in the literature to date, highlighting the extreme rarity of this condition [1].

The phenotypic spectrum of *TMX2*-related disorder continues to expand as additional patients are identified. Reported neuroimaging findings include polymicrogyria, corpus callosum abnormalities, periventricular heterotopia, and brainstem hypoplasia [1]. However, extraneurological manifestations remain poorly characterized, and congenital limb anomalies have not been systematically documented.

We report a Moroccan infant harboring a homozygous pathogenic* TMX2* variant (p.Arg205Gln) who presented with severe global developmental delay, epilepsy, progressive microcephaly, bilateral frontoparietal polymicrogyria, and bilateral second-third toe syndactyly. This observation further broadens the phenotypic spectrum associated with *TMX2*-related neurodevelopmental disorder and underscores the diagnostic utility of whole-exome sequencing in children with cortical malformations and severe neurodevelopmental impairment. This case report was conducted in accordance with the principles of the Declaration of Helsinki.

## Case presentation

Patient background and family history

The proband is a 13-month-old Moroccan male infant born to second-cousin consanguineous parents. The pregnancy was uneventful and carried to term with breech presentation. Birth weight was 3,200 g, with an immediate cry and no reported perinatal complications. No family history of similar neurological disorders, epilepsy, developmental delay, or known genetic conditions was identified. TORCH serology was negative, excluding common congenital infectious etiologies. The family pedigree is illustrated in Figure 1.

**Figure 1:**
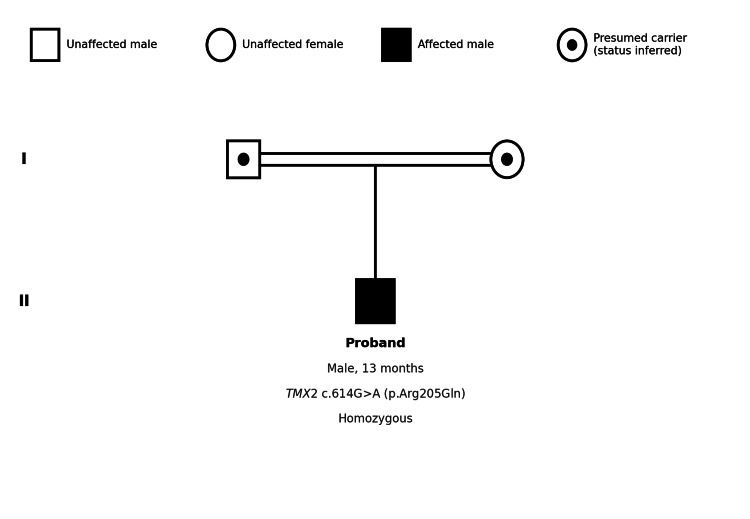
Family pedigree showing autosomal recessive inheritance of the TMX2 c.614G>A (p.Arg205Gln) variant Pedigree of the family showing a male proband affected by TMX2-related neurodevelopmental disorder carrying the homozygous c.614G>A (p.Arg205Gln) variant. Double horizontal lines indicate a second-cousin consanguineous union. Symbols with a central dot indicate presumed carrier status inferred from the autosomal recessive inheritance pattern. Parental genetic testing was not performed. Written informed consent was obtained from the patient's parents for the publication of clinical data, images, and genetic results. TMX2: thioredoxin-related transmembrane protein 2

Clinical presentation and physical examination

The patient was referred to a tertiary pediatric center for the evaluation of progressive microcephaly and severe global developmental delay. Developmental assessment revealed a complete absence of head control, inability to sit independently, inability to stand, and failure to achieve age-appropriate motor milestones. Epilepsy was diagnosed at seven months of age, manifesting as generalized tonic-clonic seizures with associated gaze fixation. Antiepileptic therapy with valproic acid was initiated with a partial clinical response.

Anthropometric measurements demonstrated severe growth impairment, with a weight of 7 kg (< -3 standard deviation (SD)), height of 65 cm (< -3 SD), and head circumference of 41 cm (< -3 SD), according to the World Health Organization (WHO) Child Growth Standards.

Physical examination revealed microcephaly, a markedly sloping forehead, bilateral strabismus, retrognathism, low-set ears, and a low anterior hairline. Neurological examination demonstrated profound axial hypotonia, absent head control, and brisk lower-limb deep tendon reflexes, suggestive of evolving spasticity.

A notable finding was bilateral cutaneous syndactyly involving the second and third toes on both feet (Figure 2), a feature not previously reported in association with *TMX2*-related neurodevelopmental disorder.

**Figure 2:**
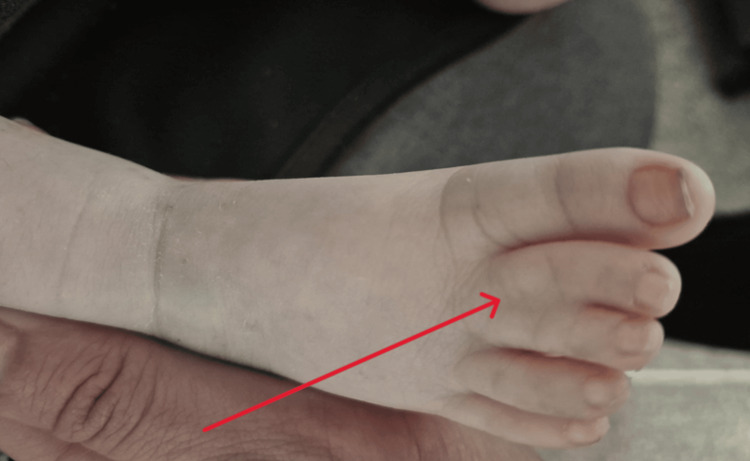
Representative image of second-third toe syndactyly Written informed consent was obtained from the patient's parents for the publication of clinical data, images, and genetic results.

Investigations

Neuroimaging

Brain magnetic resonance imaging (MRI) demonstrated bilateral frontoparietal polymicrogyria characterized by cortical thickening and irregular gyral architecture, consistent with a neuronal migration and cortical organization defect (Figure 3). Mild ventriculomegaly and prominence of the subarachnoid spaces were also observed. No periventricular heterotopia, corpus callosum abnormalities, or brainstem malformations were identified.

**Figure 3:**
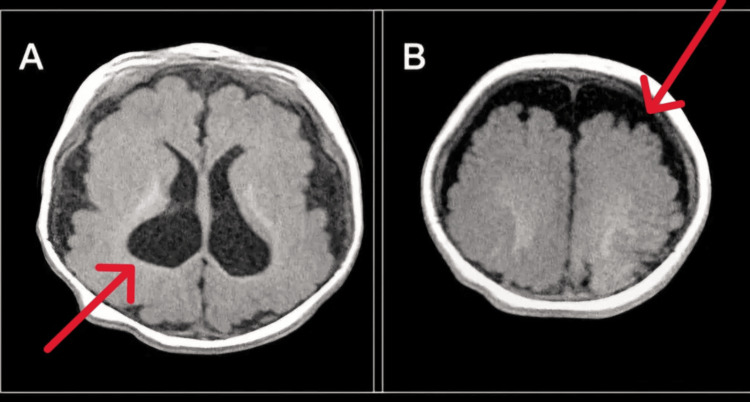
Brain MRI findings in the proband Axial T1-weighted brain MRI sequences. (A) Axial section at the level of the lateral ventricles demonstrating bilateral ventriculomegaly and prominence of the subarachnoid spaces (red arrow). (B) Axial section at the level of the frontoparietal cortex demonstrating bilateral frontoparietal polymicrogyria, characterized by cortical thickening and an irregular gyral pattern (red arrow), consistent with a neuronal migration and cortical organization defect. Written informed consent was obtained from the patient's parents for the publication of clinical data, images, and genetic results. MRI: magnetic resonance imaging

Neurophysiology

Sleep-wake electroencephalography revealed asymmetric and poorly organized background activity with the absence of physiological sleep patterns over the right hemisphere. Rare right frontotemporal and frontocentral epileptiform spike discharges were observed, occasionally becoming diffuse. Intermittent photic stimulation elicited no significant response. These findings were consistent with right hemispheric dysfunction associated with focal epileptic activity.

Additional investigations

Abdominal and renal ultrasonography was normal. Conventional karyotyping showed a normal chromosomal complement. Auditory evoked potentials were normal. Visual evoked potentials demonstrated preserved conduction along the visual pathways.

Genetic Analysis

Whole-exome sequencing was performed at a certified molecular genetics laboratory on genomic DNA extracted from peripheral ethylenediaminetetraacetic acid (EDTA) blood. Exome capture and sequencing were performed using the GeneMind Surfseq 5000 platform (GeneMind Biosciences Co., Ltd., Shenzhen, China), with alignment to the Genome Reference Consortium Human Build 38 (GRCh38). A mean depth of coverage of 132.80× was achieved across the entire genome, with 98.60% of targeted bases covered at a depth of ≥20×. Variant interpretation was performed using a proprietary bioinformatics pipeline incorporating Genome Analysis Toolkit (GATK) best practices for single nucleotide variant/insertion-deletion (SNV/INDEL) calling and American College of Medical Genetics and Genomics/Association for Molecular Pathology (ACMG/AMP) guidelines for variant classification [4].

A homozygous pathogenic missense variant was identified in *TMX2* (NM_015959.4: c.614G>A; p.Arg205Gln; genomic position chr11:57739039 G>A, GRCh38). The variant is observed at an extremely low frequency in the gnomAD v4.1.0 database (total allele frequency: 0.001%) [5] and has been previously reported as pathogenic in at least one similarly affected individual (ClinVar ID: VCV000804370; PMID: 31735293), classified as pathogenic per ACMG criteria [4]. Full variant details are summarized in Table 1.

**Table 1:** Molecular and pathogenicity characteristics of the identified TMX2 variant *TMX2:* thioredoxin-related transmembrane protein 2; ACMG: American College of Medical Genetics and Genomics; gnomAD: Genome Aggregation Database; PS3: functional studies demonstrating deleterious effect; PM2: absent from population databases; PM5: different pathogenic missense variant at the same codon previously reported (p.Arg205Trp); PP4: phenotype highly specific for* TMX2-*related neurodevelopmental disorder.

Parameter	Details
Gene	TMX2
Transcript	NM_015959.4
cDNA change	c.614G>A
Protein change	p.Arg205Gln
Genomic position (GRCh38)	chr11:57739039 G>A
Variant type	Missense
Zygosity	Homozygous
gnomAD v4.1.0 frequency	0.00%
ClinVar ID	VCV000804370
Previously reported	Yes - pathogenic (PMID: 31735293)
ACMG classification	Pathogenic
ACMG evidence codes	PS3, PM2, PM5, PP4
Disease association	Neurodevelopmental disorder with microcephaly, cortical malformations, and spasticity (OMIM: 618730)

Management and Follow-Up

Following molecular confirmation of the diagnosis, the patient was enrolled in a multidisciplinary management program including antiepileptic therapy with valproic acid, physiotherapy, developmental rehabilitation, and nutritional support. Ophthalmologic assessment was requested because of bilateral strabismus. Genetic counseling was provided to the family regarding the autosomal recessive inheritance pattern and the associated 25% recurrence risk for future pregnancies.

## Discussion

We report a Moroccan infant harboring a homozygous pathogenic *TMX2 *variant (p.Arg205Gln) presenting with the characteristic neurological phenotype of *TMX2*-related neurodevelopmental disorder, further enriched by bilateral second-third toe syndactyly, a feature not previously described in reported cases of this condition.

*TMX2* encodes a thioredoxin-related transmembrane protein localized to the ER membrane and mitochondria-associated ER membrane contact sites. Through its unique cytosolic thioredoxin domain, *TMX2* prevents the sulfenylation of mitochondrial outer membrane proteins, including TOM70, thereby regulating ER-mitochondria tethering and limiting mitochondrial calcium uptake and metabolism [2]. This molecular function is critical for maintaining ER redox homeostasis during cortical neurogenesis [1,2]. Experimental studies in zebrafish have demonstrated that loss of *TMX2* function selectively impairs neuronal survival in the developing brain, particularly in post-mitotic neurons, resulting in progressive neuronal loss and cortical malformations [3].

The *TMX2* p.Arg205Gln variant (c.614G>A) identified in our patient was previously reported by Vandervore et al. in an affected individual with a similar neurodevelopmental phenotype and is classified as pathogenic in ClinVar (VCV000804370) [1]. The affected amino acid residue is located within a highly conserved functional region of the protein, and its substitution is predicted to disrupt normal protein structure and function. The rarity of this variant in population databases further supports its pathogenicity [5].

The bilateral frontoparietal polymicrogyria observed on brain MRI is consistent with the predominant neuroimaging pattern described in previously reported *TMX2*-related cases [1]. This cortical malformation is thought to result from impaired neuronal development and abnormal cortical organization secondary to disrupted ER homeostasis during critical stages of brain development [1,3]. Polymicrogyria is a highly epileptogenic malformation associated with diverse genetic etiologies, and epilepsy is a frequent feature among affected individuals [6,7]. The electroencephalographic abnormalities observed in our patient, including right hemispheric dysfunction and focal epileptiform discharges, are consistent with the underlying cortical malformation and mirror findings reported in other affected patients [1].

A notable finding in our patient is the presence of bilateral cutaneous syndactyly involving the second and third toes. To our knowledge, this feature has not been described in previously reported *TMX2*-related cases. Although isolated second-third toe syndactyly may occur as a minor developmental variant in the general population, its occurrence in the setting of a genetically confirmed *TMX2-*related disorder raises the possibility of an expanded phenotypic spectrum. Further reports will be necessary to determine whether syndactyly represents a recurrent extraneurological manifestation or an incidental finding.

This case underscores the diagnostic value of whole-exome sequencing in infants presenting with cortical malformations, epilepsy, and severe neurodevelopmental delay, particularly in consanguineous populations, where diagnostic yields are markedly higher than in non-consanguineous cohorts [8]. Early molecular diagnosis facilitates accurate genetic counseling, improves prognostic assessment, and allows implementation of appropriate multidisciplinary management strategies for affected patients and their families.

## Conclusions

We report a Moroccan infant with *TMX2*-related neurodevelopmental disorder caused by a homozygous pathogenic p.Arg205Gln variant, presenting with bilateral frontoparietal polymicrogyria, severe global developmental delay, early-onset epilepsy, and bilateral second-third toe syndactyly. The latter represents a previously undescribed feature that may broaden the recognized phenotypic spectrum of this rare disorder, although confirmation in additional patients will be required.

This case highlights the diagnostic utility of whole-exome sequencing in infants presenting with cortical malformations and severe neurodevelopmental impairment, particularly in consanguineous families in whom autosomal recessive disorders are more prevalent. Early molecular diagnosis is essential for accurate genetic counseling, prognostic assessment, and implementation of appropriate multidisciplinary management strategies.
